# Supplementary material for: Impact of Preoperative Malnutrition on Postoperative Quality of Life in Older Adults Undergoing Surgery for Degenerative Cervical Myelopathy: A Retrospective Cohort Study
Source: Nutrients. 2025 Sep 9;17(18):2912. doi: 10.3390/nu17182912 (PMC12472808; doi:10.3390/nu17182912)
Supplement: Supplementary file 1 [file nutrients-17-02912-s001.zip › nutrients-3852086-supplementary.pdf]

Supplementary Table S1. Multivariate analyses of EQ-5D score deterioration exceeding the MCID of -0.0485 at one year postoperatively, with age excluded from the covariates in a sensitivity analysis.

	Odds ratio [CI]	P
Sex		0.86
Male	1.06 [0.53–2.17]	
Female	Reference	
Preoperative GNRI		0.058
≤ 98	2.20 [0.97–4.87]	
> 98	Reference	
Incidental durotomy		0.07
Yes	< 0.001 [*-1.18]	
No	Reference	

EQ-5D, EuroQol five-dimension questionnaire; CI, confidence interval; GNRI, Geriatric Nutritional Risk Index

Supplementary Table S2. Multivariate analyses of EQ-5D score deterioration exceeding the MCID of -0.0485 at one year postoperatively, with sex excluded from the covariates in a sensitivity analysis.

	Odds ratio [CI]	P
Age, years		0.72
85-	0.68 [0.13–2.70]	
75–84	1.18 [0.57–2.46]	
65–74	Reference	
Preoperative GNRI		**0.04**
≤ 98	2.39 [1.03–5.47]	
> 98	Reference	
Incidental durotomy		0.06
Yes	< 0.001 [*-1.15]	
No	Reference	

EQ-5D, EuroQol five-dimension questionnaire; CI, confidence interval; GNRI, Geriatric Nutritional Risk Index

Supplementary Table S3. Multivariate analyses of EQ-5D score deterioration exceeding the MCID of -0.0485 at one year postoperatively, with incidental durotomy excluded from the covariates in a sensitivity analysis.

	Odds ratio [CI]	P
Age, years		0.72
85-	0.65 [0.13–2.50]	
75–84	1.13 [0.55–2.38]	
65–74	Reference	
Sex		0.75
Male	1.12 [0.56–2.30]	
Female	Reference	
Preoperative GNRI		**0.04**
≤ 98	2.39 [1.04–5.42]	
> 98	Reference	

EQ-5D, EuroQol five-dimension questionnaire; CI, confidence interval; GNRI, Geriatric Nutritional Risk Index
